# A Deceptively Quiet Case of Gangrenous Cholecystitis

**DOI:** 10.7759/cureus.111324

**Published:** 2026-06-22

**Authors:** Rachel M Johnson, Greta L Becker

**Affiliations:** 1 Medicine, University of Iowa, Iowa City, USA; 2 Internal Medicine, University of Iowa, Iowa City, USA

**Keywords:** acute cholecystitis, acute gangrenous cholecystitis, diabetes mellitus in adults, endoscopic retrograde cholangiopancreatography (ercp), gall blader disease

## Abstract

Acute gangrenous cholecystitis is a severe complication of acute cholecystitis. We report a 70-year-old male with type 1 diabetes mellitus who presented with intractable vomiting without abdominal pain. CT imaging showed gallbladder wall thickening and concern for gallbladder perforation. Patient underwent a laparoscopic cholecystectomy, which revealed a gangrenous gallbladder with surrounding purulent drainage. The patient recovered after an extended hospital course. This case highlights the importance of considering atypical presentations of severe intra-abdominal pathology in diabetic patients.

## Introduction

Acute cholecystitis (AC), or inflammation of the gallbladder, occurs most commonly from obstruction of the cystic duct by gallstones or biliary sludge. The onset of AC is generally preceded by an episode of biliary colic. Patients typically present with right upper quadrant abdominal pain that may radiate to the back, a positive sonographic Murphy’s sign, nausea, vomiting, and fever. Elevated bilirubin, white blood cell count, and C-reactive protein are common laboratory findings [1]. The Tokyo Guidelines 2018 provide standardized diagnostic criteria for AC based on systemic signs of inflammation, evidence of cholestasis, and imaging findings, and are widely used to guide diagnosis and severity grading [2].

Acute gangrenous cholecystitis (AGC) is a complication of AC that occurs when the gallbladder wall undergoes necrosis [1]. Of patients with AC, up to 30% will develop AGC [3]. Factors associated with AGC include male sex, diabetes mellitus, cardiovascular disease, and delayed hospital presentation. A feared complication of AGC is gallbladder perforation, which has mortality rates of up to 30% [3, 4]. Perforation of the gallbladder wall can lead to a transient decrease in abdominal pain, followed by the onset of peritonitis [4]. In this case report, we present an atypical case of perforated gangrenous cholecystitis in an adult male with diabetes mellitus.

## Case presentation

A 70-year-old male with past medical history of late onset type 1 diabetes mellitus and hypertension presented to the emergency department with a chief complaint of intractable vomiting for two weeks in duration. He was unable to tolerate oral intake or medications at home. Despite this acute illness, blood glucose levels were controlled with his insulin pump. He denied any associated abdominal pain or fevers.

Vital signs on arrival were within normal limits. His abdomen was non-tender to palpation, with no guarding or rebound. Initial lab values are listed in Table 1.

**Table 1:** Laboratory values at initial presentation Reference ranges are provided for comparison. WBC: white blood cell count; Hgb: hemoglobin; ALT: alanine aminotransferase; AST: aspartate aminotransferase; ALP: alkaline phosphatase; HCO3: bicarbonate; CRP: C-reactive protein; BHB: beta-hydroxybutyrate.

	Value	Reference	units
WBC	9.7	4.0–11.0	K/uL
Hgb	12.8	13.0–17.0	g/dL
ALT	51	7–56	U/L
AST	54	10–40	U/L
ALP	80	44–147	U/L
Total bilirubin	0.6	0.1–1.2	mg/dL
Albumin	3.5	3.4–5.4	g/dL
HCO3	27.4	22–29	mmol/L
Glucose	143	70–99	mg/dL
Lactic acid	1	0.5–2.0	mmol/L
CRP	206.2	<10	mg/L
BHB	2.2	0.0–0.5	mEq/L

Laboratory evaluation demonstrated a white blood cell count of 9.7 K/μL, blood glucose level 143 mg/dL, bicarbonate 27.4 mmol/L, alanine aminotransferase 51 U/L, aspartate aminotransferase 54 U/L, and C-reactive protein of 206.2 mg/L.

He was admitted for observation and managed supportively with fluid replacement and antiemetics. On hospital day one, he continued to have intractable emesis. He transitioned off his home insulin pump to basal and sliding-scale insulin. His blood glucose levels were difficult to control after removal of the pump, with levels ranging from 100 to over 400 mg/dL.

An abdominal computed tomography (CT) scan was obtained, which showed a hydropic gallbladder, pericholecystic stranding, mild gallbladder wall thickening, and an indistinct border between the gallbladder and liver (Figure 1).

**Figure 1:**
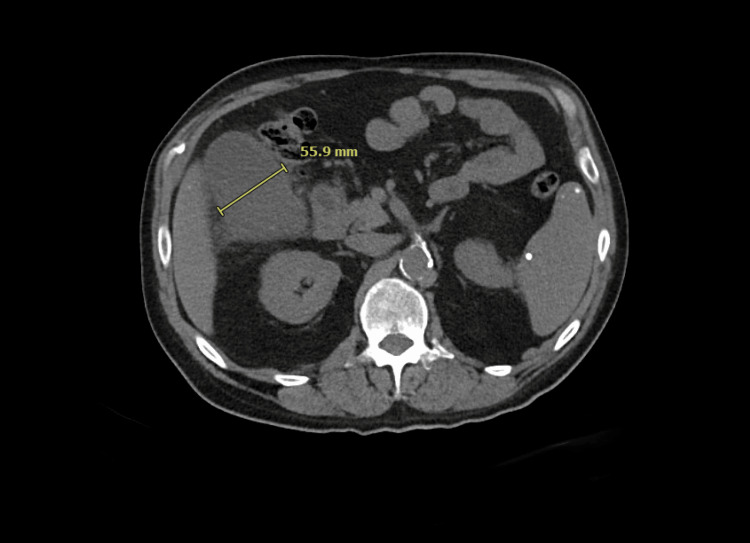
CT abdomen at initial presentation Axial non-contrast abdominal computed tomography (CT) image obtained at the time of initial presentation showing a hydropic gallbladder measuring approximately 5.6 cm with mild gallbladder wall thickening and an indistinct border between the gallbladder and liver parenchyma concerning for complicated acute cholecystitis.

Abdominal ultrasound showed a distended gallbladder with sludge with possible perforation (Figure 2).

**Figure 2:**
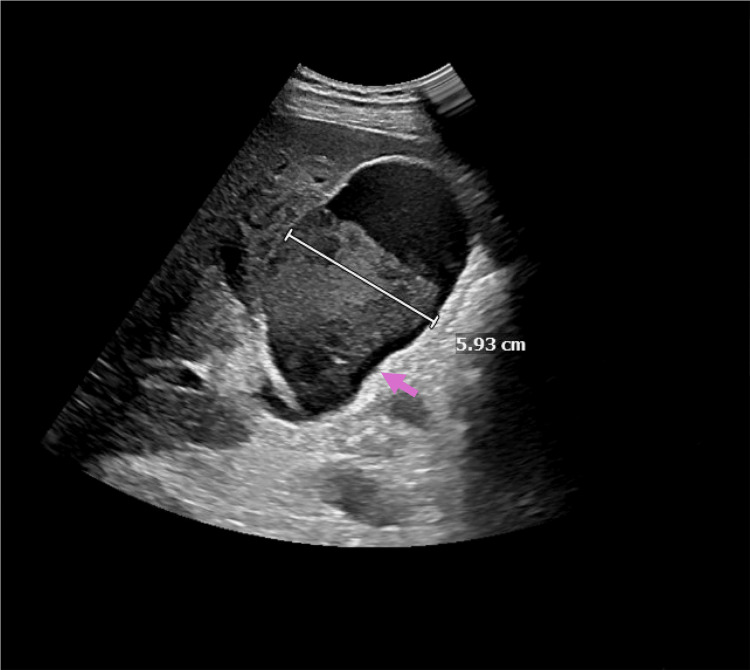
Abdominal ultrasound at initial presentation Abdominal ultrasound at time of initial presentation demonstrating a diffusely distended gallbladder measuring approximately 5.93 cm with sludge and a potential gallbladder wall defect (pink arrow).

The patient was started on piperacillin-tazobactam and underwent diagnostic laparoscopy.

During surgery, the anterior wall of the gallbladder appeared gangrenous with areas of walled-off necrosis and purulent drainage. There appeared to be an abscess with liquefactive necrosis extending into the liver. The cystic duct was surgically closed, and a subtotal cholecystectomy was performed with the resection of the anterior wall of the gallbladder from the liver bed. A drain was placed at the cystic duct stump site and left in place post-operatively for additional source control. Pathology of the gallbladder revealed acute necrotizing cholecystitis. 

Within twenty-four hours postoperatively, the patient developed new abdominal pain, recurrent nausea, and fevers of 39°C. He was tachycardic with a heart rate of 107 beats per minute, and white count was elevated to 30.4 K/uL. Due to concern for sepsis, antibiotics were broadened to meropenem and vancomycin. A repeat abdominal CT scan obtained on postoperative day two demonstrated a fluid collection within the gallbladder fossa, and the patient was noted to have increasing bilious drain output, raising concern for a postoperative bile leak (Figure 3).

**Figure 3:**
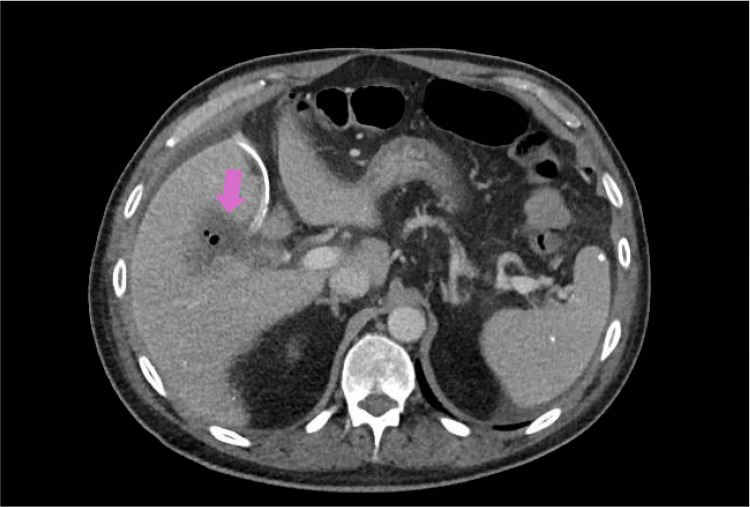
CT abdomen post-cholecystectomy Axial contrast-enhanced abdominal computed tomography (CT) demonstrating postoperative changes status post cholecystectomy, with a focal fluid collection (pink arrow) and air bubbles within the gallbladder fossa.

The patient underwent successful endoscopic retrograde cholangiopancreatography (ERCP), which confirmed a bile leak via the cystic duct remnant (Figure 4), and a bile duct stent was successfully placed.

**Figure 4:**
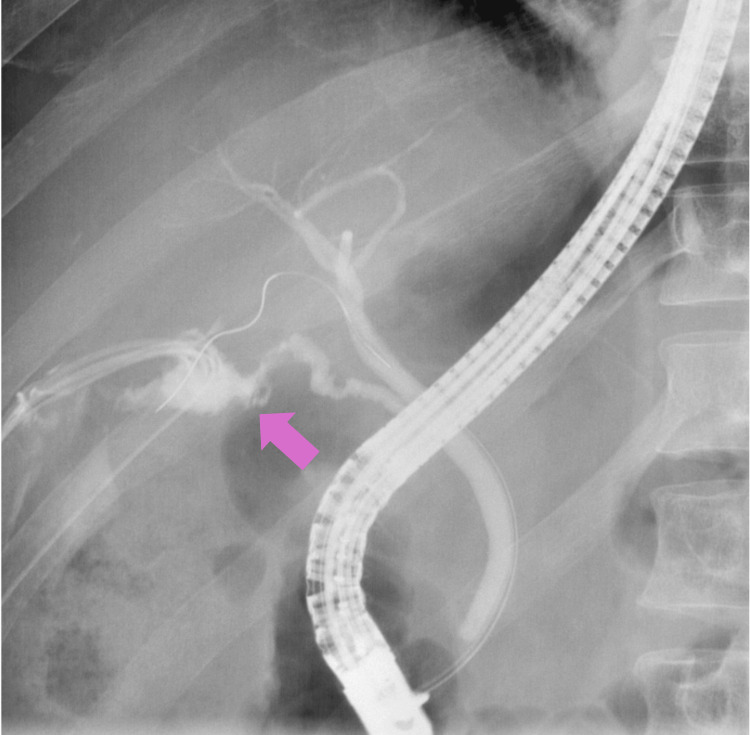
ERCP demonstrating post-operative bile leak Endoscopic retrograde cholangiopancreatography (ERCP) showing contrast extravasation (pink arrow) from the subtotal cholecystectomy stump, consistent with a postoperative bile leak.

Following the ERCP, the patient’s pain and nausea improved. Antibiotics were narrowed to amoxicillin-clavulanic acid, the biliary drain was removed, and he was discharged on hospital day 11. At a one-week hospital follow-up appointment, the patient was doing well without nausea or abdominal pain, and laboratory work was within normal limits. 

## Discussion

The classic presentation of AC involves right upper quadrant abdominal pain, fevers, and nausea. This case demonstrated a patient with severe nausea but an otherwise “silent abdomen”. The presentation of AC in diabetic patients has been relatively underexplored. One study found an association between type 2 diabetes mellitus and absent abdominal pain among elderly patients presenting with an acute abdomen [5]. Similarly, there have been multiple cases of painless myocardial infarction and one case of painless upper limb ischemia, reported in diabetic populations [3, 6].

The silent abdomen in our patient may have been related to diabetic neuropathy, resulting in blunted pain perception. Prolonged hyperglycemia causes increased flux through glucose metabolic pathways. This is thought to increase the production of reactive oxygen species and advanced glycation end products, increase cellular osmolality and oxidative stress, and trigger long-term inflammatory responses. Together, these mechanisms lead to neuronal cell damage and neuropathy. This neuropathy is commonly known to blunt peripheral pain sensation but can also affect visceral pain sensation [7].

Diabetes mellitus has been implicated as a risk factor for the development of AGC, likely due to the strong association between diabetes and atherosclerotic disease. Cystic artery atherosclerosis and microvascular disease can lead to gallbladder ischemia and gangrenous transformation [8]. 

The diagnosis of AC includes a combination of clinical symptoms and imaging findings. Current guidelines recommend ultrasound as the first imaging modality for AC due to its accuracy, ability to detect gallstones, cost, portability, and timeliness. If ultrasound is non-diagnostic, an abdominal CT with intravenous contrast should be performed [9]. CT is more favorable than US for the diagnosis of complicated AC, including the detection of complex abscesses or bowel pathology [10]. US findings that are associated with AGC include a thickened gallbladder wall and pericholecystic fluid [11]. CT findings of AGC include irregular gallbladder wall thickening and decreased enhancement of the gallbladder wall after contrast is administered [12]. Definitive diagnosis of AGC requires histopathological analysis showing necrosis of the gallbladder wall [13].

The ability to distinguish AGC from non-gangrenous AC is important, as recommendations for surgical timing differ. Current guidelines recommend laparoscopic cholecystectomy within 10 days of symptom onset for non-complicated acute calculous cholecystitis versus emergent surgery for patients with severe complications, including AGC [14].

## Conclusions

In this case report, we describe an adult male with AGC who presented with intractable vomiting but no abdominal pain or tenderness. This case underscores the importance of maintaining a high index of suspicion for abdominal pathology in patients with diabetes mellitus, as serious diseases like AGC may present atypically. 
